# MRI for Acute Pelvic Pain in Pediatric Females After Inconclusive Ultrasound: Diagnostic Performance of Non-Contrast-Enhanced and Contrast-Enhanced Protocols

**DOI:** 10.3390/diagnostics16060912

**Published:** 2026-03-19

**Authors:** Gabriele Masselli, Giacomo Bonito, Laura Corso, Silvia Gigli, Lucia Malzone, Paolo Ricci

**Affiliations:** 1 Department of Emergency Radiology, Policlinico Umberto I Hospital, Sapienza University of Rome, Viale del Policlinico 155, 00161 Rome, Italy; gabriele.masselli@uniroma1.it (G.M.); paolo.ricci@uniroma1.it (P.R.); 2 Department of Radiological, Oncological and Pathological Sciences, Policlinico Umberto I Hospital, Sapienza University of Rome, Viale Regina Elena 324, 00161 Rome, Italy; laura.corso@uniroma1.it (L.C.); lucia.malzone@uniroma1.it (L.M.); 3 Department of Diagnostic Imaging, Sandro Pertini Hospital, Via dei Monti Tiburtini 385, 00157 Rome, Italy; silvia.gigli@aslroma2.it

**Keywords:** acute pelvic pain, magnetic resonance imaging, pediatric radiology, contrast-enhanced MRI, ultrasonography

## Abstract

**Background/Objectives**: Acute pelvic pain in pediatric female patients is a common presentation in the emergency setting and poses significant diagnostic challenges. Ultrasonography is the first-line imaging modality, but a substantial proportion of examinations remain inconclusive. Magnetic resonance imaging (MRI) is increasingly used as a second-line modality in this context; however, evidence comparing the diagnostic performance of non-contrast-enhanced and contrast-enhanced MRI protocols in pediatric patients remains limited. The aim of this study was to assess the diagnostic accuracy of MRI in pediatric females with acute pelvic pain after inconclusive ultrasonography and to compare non-contrast-enhanced and contrast-enhanced MRI protocols. **Methods**: This single-center observational study included pediatric female patients presenting with acute pelvic pain who underwent MRI after inconclusive ultrasonography. MRI examinations were performed using a standardized protocol including non-contrast-enhanced sequences and diffusion-weighted imaging. Administration of contrast material was determined by the attending radiologist according to clinical indications. Three radiologists with different levels of experience independently reviewed all examinations in two separate reading sessions (without and with contrast). Diagnostic performance, interobserver and intraobserver agreement, diagnostic confidence, and examination duration were assessed using a composite clinical reference standard. **Results**: Eighty-eight patients (mean age, 13.5 years; range, 7–17 years) were included. MRI identified a specific cause of acute pelvic pain in 60 patients (68.2%), with gynecological conditions accounting for 75.0% of positive findings. Hemorrhagic ovarian cysts and adnexal torsion were the most frequent diagnoses. Both non-contrast-enhanced and contrast-enhanced MRI demonstrated high diagnostic accuracy across all readers, with no statistically significant differences in sensitivity or specificity between protocols (*p* > 0.05). Contrast-enhanced MRI was associated with higher diagnostic confidence for all readers (*p* < 0.001) but longer examination times. **Conclusions**: MRI is a reliable second-line imaging modality for evaluating acute pelvic pain in pediatric female patients after inconclusive ultrasonography. Non-contrast-enhanced MRI combined with diffusion-weighted imaging provides robust diagnostic performance in most cases, while contrast-enhanced MRI may be reserved for selected equivocal cases.

## 1. Introduction

Acute pelvic pain (APP) is a frequent cause of emergency department visits among pediatric patients. Overall, up to 10% of pediatric emergency department presentations are related to abdominal pain; among female patients, acute pelvic pain represents one of the most common complaints, accounting for approximately 20% of laparoscopic procedures and 2–10% of outpatient gynecological consultations [1,2].

In pediatric females, APP poses a substantial diagnostic challenge. Clinical history and physical examination are often limited, and the differential diagnosis is broad, encompassing both gynecological and non-gynecological conditions. Gynecological causes include uterine, tubal, and ovarian pathologies, whereas non-gynecological etiologies may arise from gastrointestinal, urogenital, or vascular diseases [3]. Prompt and accurate identification of the underlying cause is essential to guide appropriate management, particularly to distinguish surgical emergencies from conditions amenable to conservative treatment.

Given the complexity of APP in the pediatric population, imaging plays a central role in diagnostic evaluation and clinical decision-making. Ultrasonography (US), including color Doppler imaging, is widely accepted as the first-line imaging modality because of its noninvasiveness and widespread availability [4]. However, US has several limitations that may reduce diagnostic accuracy, including patient obesity, bowel gas, and limited patient cooperation due to pain. Moreover, US is highly operator dependent and is optimally performed by experienced pediatric sonographers.

In pediatric patients, transabdominal suprapubic US is the only feasible approach, as transvaginal imaging is not appropriate. Although computed tomography (CT) provides excellent anatomic detail and is highly effective for the evaluation of non-gynecological conditions such as appendicitis and urolithiasis, its role in assessing gynecological pathology is limited. In addition, concerns regarding ionizing radiation exposure are particularly relevant in the pediatric population [3,4].

Magnetic resonance imaging (MRI) offers superior soft-tissue contrast resolution and detailed anatomic delineation without the use of ionizing radiation, making it particularly well suited for pelvic imaging in children and adolescents. MRI has emerged as a reliable imaging modality in the emergency and urgent care settings for differentiating gynecological from non-gynecological causes of APP in female pediatric patients with inconclusive US findings [5,6]. Recent reports have documented an increasing use of MRI in pediatric emergency departments, particularly for the evaluation of abdominal and pelvic pain in female patients [7].

Despite growing evidence supporting the diagnostic value of MRI, data remain limited regarding its performance in the comprehensive assessment of acute pelvic pain in pediatric females, especially when comparing unenhanced and contrast-enhanced MRI protocols.

Therefore, the aims of this observational study were: (1) to evaluate the diagnostic accuracy of MRI in the assessment of acute pelvic pain in the pediatric female population following inconclusive ultrasonography; and (2) to compare the diagnostic performance of unenhanced versus contrast-enhanced MRI protocols.

## 2. Materials and Methods

### 2.1. Patients

This study represents a single-center observational cohort based on prospectively acquired MRI examinations performed as part of routine emergency clinical care, with subsequent retrospective analysis of anonymized imaging data for research purposes.

All examinations were performed in accordance with the ethical standards of the local ethics committee on human experimentation. Informed consent and assent were obtained in accordance with institutional clinical practice at the time of MRI performance, and imaging examinations were not modified or influenced by study participation.

The inclusion criteria comprised pediatric female patients presenting to the emergency department with acute pelvic pain (APP) who underwent magnetic resonance imaging (MRI) following inconclusive ultrasonography (US). Ultrasonography was considered inconclusive when it failed to identify a definite gynecological or non-gynecological cause of acute pelvic pain, or when imaging findings were discordant with clinical and laboratory data and did not adequately explain the patient’s presentation.

Exclusion criteria included claustrophobia, inability to cooperate with MRI acquisition, and contraindications to MRI, such as known allergy to gadolinium-based contrast agents or severe renal impairment (estimated glomerular filtration rate < 30 mL/min).

Between January 2019 and June 2025, a total of 503 consecutive pediatric female patients presenting with APP were evaluated in the emergency department of our institution and underwent initial US examination. In 408 cases (81.1%), the combination of US findings and laboratory results allowed a conclusive diagnosis. In the remaining 95 cases (18.9%), US findings were considered indeterminate.

Among these 95 patients, seven (7.4%) did not undergo MRI: four patients (4.2%) were unable to remain still because of severe pain, and three unstable patients (3.1%) were referred directly for surgical intervention. The remaining 88 patients (92.6%) underwent emergency MRI for further diagnostic evaluation and were subsequently included in the study population. The mean age of the included patients was 13.5 years (range, 7–17 years).

**Figure 1:**
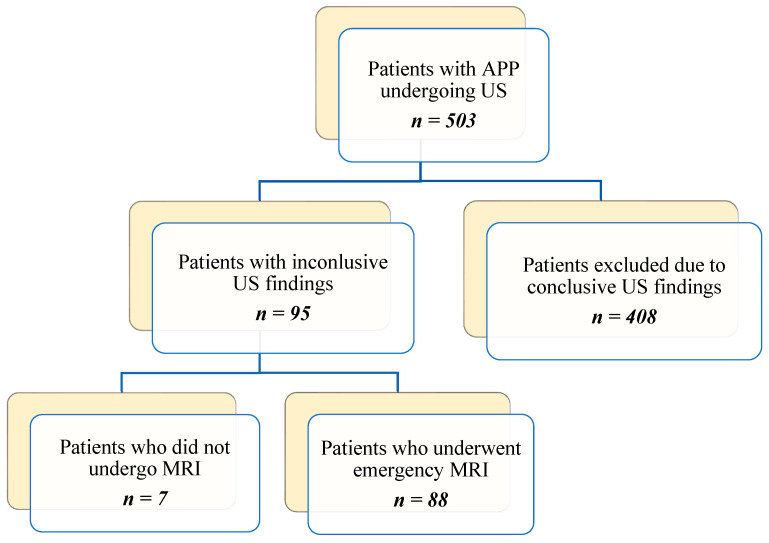
Flowchart of patients inclusion/exclusion process in the study.

The patient inclusion and exclusion process is summarized in Figure 1.

### 2.2. MRI Protocol

MRI examinations were performed using a 1.5-T scanner (Magnetom Sola; Siemens Healthineers, Erlangen, Germany) equipped with advanced gradient systems (maximum gradient strength, 45 mT/m; slew rate, 200 T/m/s). A 32-channel phased-array body coil was used for image acquisition. All patients were examined in the supine position.

None of the patients required sedation or general anesthesia during MRI examinations, as all included patients were cooperative and able to tolerate the imaging procedure.

T2-weighted images were acquired in the axial and coronal planes using single-shot half-Fourier rapid acquisition with relaxation enhancement (RARE) sequences (half-Fourier acquisition single-shot turbo spin-echo [HASTE]). High-resolution T2-weighted fast spin-echo (FSE) sequences were obtained in the axial and sagittal planes.

Diffusion-weighted imaging (DWI) was performed using a single-shot spin-echo echo-planar imaging (EPI) sequence with fat suppression and parallel imaging. Acquisition parameters included a repetition time/echo time of 2500/80 ms, matrix size of 128 × 128, field of view of 280 mm × 400 mm, section thickness of 6 mm with a 1 mm intersection gap, six signal averages, and a receiver bandwidth of 1930 Hz/pixel. DWI was acquired using b values of 50, 500, and 1000 s/mm^2^, with a total acquisition time of approximately 3 min.

Apparent diffusion coefficient (ADC) maps were automatically generated using commercially available software (Leonardo workstation; Siemens Healthineers, Erlangen, Germany) by combining all b values and averaging the three orthogonal diffusion directions.

To reduce bowel motion artifacts, scopolamine N-butylbromide (0.3 mg/kg) was administered intravenously before image acquisition after exclusion of contraindications [6]. A baseline axial T1-weighted fat-saturated ultrafast gradient-recalled echo sequence was then obtained.

For contrast-enhanced imaging, a gadolinium-based contrast agent (gadoteridol, 0.1 mmol/kg) was administered intravenously at a rate of 2 mL/s, followed by a saline flush. Dynamic three-dimensional T1-weighted gradient-recalled echo sequences with fat saturation were acquired in the axial plane during the arterial and portal venous phases. A board-certified radiologist supervised the examination and modified the protocol when necessary. MRI examinations were interpreted during the same emergency department visit, and the final radiology report was typically issued within the same clinical encounter. The administration of gadolinium-based contrast agents was determined exclusively by the attending radiologist on clinical grounds, in accordance with institutional emergency imaging protocols, and was not mandated by the study design. All images were acquired as part of routine clinical MRI examinations. Minimal post-processing was applied uniformly to the entire image for visualization purposes only (windowing and level adjustment), without altering the original data.

The detailed MRI acquisition parameters are summarized in Table 1.

### 2.3. Imaging Analysis

All MRI examinations were independently reviewed by three radiologists with different levels of experience in pelvic MRI (20, 10, and 5 years, respectively). Prior to image interpretation, predefined diagnostic categories were established based on clinically relevant and commonly encountered causes of acute pelvic pain, in order to ensure consistency and standardization across cases.

Causes of acute pelvic pain were classified as gynecological or non-gynecological, in accordance with previously published classifications [1,2,3,4,5,6,7,8]. Within the gynecological category, the following groups were defined: bleeding (including hemorrhagic corpus luteum and ruptured or hematoma-containing adnexal cysts); adnexal torsion (including ovarian torsion associated with cysts, teratomas, or other masses, as well as isolated tubal torsion); inflammatory conditions (pelvic inflammatory disease, tubo-ovarian abscess, and cervicitis); pelvic masses; and congenital anomalies.

Non-gynecological causes were subdivided into gastrointestinal (appendicitis, inflammatory bowel disease, infectious enteritis, bowel obstruction or perforation, and complications of Meckel diverticulum), urological (urolithiasis and complicated urinary tract infections), and musculoskeletal conditions.

To minimize recall bias, image interpretation was performed in two separate reading sessions spaced four weeks apart. All patient identifiers and clinical information were removed from the imaging datasets prior to review. During the first session, readers evaluated only non-contrast-enhanced images, including diffusion-weighted imaging. During the second session, all sequences, including post-contrast-enhanced images, were available for interpretation.

For each examination, readers independently identified and characterized the presumed cause of acute pelvic pain according to the predefined diagnostic categories. The likelihood of disease presence was graded using a five-point confidence scale (0 = definitely absent, 1 = probably absent, 2 = possibly present, 3 = probably present, 4 = definitely present).

Following an interval of three weeks, a third consensus reading was performed to resolve discrepancies among readers. The final consensus diagnosis was used for comparison with the reference standard.

### 2.4. Reference Standard

The reference standard was established through a comprehensive review of medical records for all included patients. Data collected included clinical management (surgical intervention, medical treatment, or observation), final diagnosis at discharge, and histopathological findings when available.

Of the 88 patients included in the study, 27 (30.7%) underwent surgical intervention, 13 (14.8%) received medical treatment, and 48 (54.5%) were managed conservatively with clinical observation and follow-up. MRI findings were compared with these clinical, surgical, and pathological data to assess diagnostic accuracy and to validate imaging-based diagnoses. Among surgically treated patients, imaging findings were confirmed by intraoperative findings and histopathological examinations, when available. In the remaining patients managed conservatively, the final diagnosis was established on the basis of clinical evolution and follow-up. Although surgical or histopathologic confirmation was not available in all cases, this composite reference standard reflects routine pediatric emergency practice, where conservative management and clinical follow-up are frequently adopted.

### 2.5. Statistical Analysis

For each reader, the diagnostic performance of MRI without and with contrast agent was assessed by calculating sensitivity, specificity, positive predictive value (PPV), negative predictive value (NPV), and overall accuracy in identifying the cause of acute pelvic pain in pediatric patients. Clinical follow-up, surgical findings, and histopathologic results, when available, served as the reference standard.

Differences in diagnostic performance between non-contrast-enhanced and contrast-enhanced MRI protocols were evaluated using the McNemar test. Interobserver agreement between the senior reader and the intermediate and junior readers was assessed separately for non-contrast-enhanced and contrast-enhanced MRI using Cohen’s κ statistics. Intraobserver agreement between interpretations performed without and with contrast agent was also evaluated for each reader using Cohen’s κ.

A dependent-samples *t* test was used to assess whether diagnostic confidence scores differed significantly between MRI examinations performed without and with contrast agent. The same test was applied to compare examination duration between the two protocols. Diagnostic confidence scores were reported as mean values with standard deviations.

Statistical significance was defined as a *p* value less than 0.05. All statistical analyses were performed using commercially available software packages, including Statistical Package for the Social Sciences (SPSS Statistics, version 25.0; IBM, Chicago, IL, USA), GraphPad Prism (version 5.02; GraphPad Software, San Diego, CA, USA), and MedCalc (version 13.0.2; MedCalc Software, Ostend, Belgium).

Agreement strength was interpreted according to established κ value thresholds: values of 0.20 or less indicated poor agreement; values of 0.21–0.40, fair agreement; values of 0.41–0.60, moderate agreement; values of 0.61–0.80, good agreement; and values greater than 0.80, excellent agreement. Ninety-five percent confidence intervals were calculated using the Clopper–Pearson exact method.

## 3. Results

### 3.1. Study Population and MRI Examinations

A total of 88 pediatric female patients underwent MRI examinations performed both without and with contrast agent. The mean age of the study population was 13.5 years (range, 7–17 years). At MRI, 60 of 88 patients (68.2%) were diagnosed with a specific cause of acute pelvic pain, whereas 28 patients (31.8%) showed no imaging evidence of an underlying acute condition. Of the 88 included patients, 27 (30.7%) underwent surgical intervention, 13 (14.8%) received medical treatment, and 48 (54.5%) were managed conservatively with clinical observation and follow-up. Most surgically treated cases corresponded to adnexal torsion and appendicitis, which represent the main surgical emergencies in this clinical setting. Conversely, most hemorrhagic cyst ruptures and inflammatory gynecological conditions were managed conservatively.

### 3.2. Distribution of Final Diagnoses

#### 3.2.1. Gynecological Causes

Gynecological causes accounted for 45 of 60 positive cases (75.0%).

Bleeding was the most frequent gynecological diagnosis, observed in 23 of 60 patients (38.3%). Specifically:

- Hemorrhagic ovarian cysts were identified in 19 patients (31.6%);
- Ovarian rupture was observed in four patients (6.7%).

Adnexal torsion was diagnosed in 13 of 60 patients (21.7%), including:

- Ovarian torsion associated with cysts in nine cases (15.0%);
- Ovarian torsion associated with teratomas in three cases (5.0%);
- Isolated tubal torsion in one case (1.7%).

Inflammatory gynecological conditions accounted for five cases (8.3%), including:

- Pelvic inflammatory disease in three patients (5.0%);
- Cervicitis in one patient (1.7%);
- Tubo-ovarian abscess in one patient (1.7%).

Pelvic masses were detected in four patients (6.7%), including:

- Teratomas in two cases (3.3%);
- Uterine fibroids in one case (1.7%);
- Malignancy in one case (1.7%).

#### 3.2.2. Non-Gynecological Causes

Non-gynecological causes were identified in 15 of 60 positive cases (25.0%).

Gastrointestinal inflammatory conditions accounted for 10 cases (16.7%), including:

- Appendicitis in six patients (10.0%);
- Meckel diverticulitis in one patient (1.7%);
- Crohn’s disease in one patient (1.7%);
- Ulcerative colitis in one patient (1.7%);
- Epiploic appendagitis in one patient (1.7%).

Intestinal obstruction was diagnosed in two cases (3.3%), one of which was related to congenital malformations.

Urological causes were identified in two cases (3.3%), both related to pyelonephritis.

Musculoskeletal pathology was the least frequent cause and was represented by one case of pubic osteomyelitis (1.7%).

The distribution of final clinical diagnoses is summarized in Table 2 and Table 3.

### 3.3. Diagnostic Performance of MRI

Overall, MRI demonstrated high sensitivity and specificity for both non-contrast-enhanced and contrast-enhanced protocols in identifying the cause of acute pelvic pain (*p* < 0.001).

#### 3.3.1. Non-Contrast-Enhanced MRI

Using the non-contrast-enhanced protocol, three false-negative findings were observed for all readers, related to active bleeding (two ovarian hemorrhages and one case of Meckel diverticulitis). In addition, the junior reader failed to identify two further conditions, including one case of epiploic appendagitis and one case of cervicitis. All discrepancies were resolved during the consensus review.

The diagnostic performance of non-contrast-enhanced MRI is summarized in Table 4.

The mean duration of MRI examinations performed without contrast agent was 16.1 min (range, 15–20 min).

#### 3.3.2. Contrast-Enhanced MRI

The diagnostic performance of contrast-enhanced MRI is summarized in Table 5.

The mean duration of contrast-enhanced MRI examinations was 22.5 min (range, 21–25 min).

The McNemar test did not demonstrate statistically significant differences between non-contrast-enhanced and contrast-enhanced MRI for any of the three readers (*p* > 0.05).

### 3.4. Diagnostic Confidence and Reader Agreement

Mean diagnostic confidence scores without and with contrast agent were:

- Senior reader: 4.27 and 4.80;
- Intermediate reader: 4.27 and 4.75;
- Junior reader: 3.33 and 4.49.

The dependent-samples *t* test demonstrated that diagnostic confidence scores were significantly higher for contrast-enhanced MRI across all readers. The mean differences were 0.531 (SD = 0.767) for the senior reader, 0.473 (SD = 0.573) for the intermediate reader, and 1.163 (SD = 1.264) for the junior reader (*p* < 0.001).

Intraobserver agreement between interpretations performed without and with contrast agent was excellent for the senior reader (κ = 0.925) and intermediate reader (κ = 0.881) and good for the junior reader (κ = 0.761).

Interobserver agreement for non-contrast-enhanced MRI between the senior reader and the intermediate and junior readers was good (κ = 0.695 and κ = 0.616, respectively). For contrast-enhanced MRI, interobserver agreement improved and was good to excellent, with κ values of 0.768 and 0.750, respectively.

## 4. Discussion and Conclusions

Acute pelvic pain in pediatric female patients represents a complex diagnostic scenario due to the wide spectrum of possible gynecological and non-gynecological etiologies and the frequent limitations of clinical evaluation. Although ultrasonography remains the first-line imaging modality, its diagnostic performance may be suboptimal in a relevant proportion of cases, particularly when patient-related or technical factors limit image quality.

In this study, we demonstrated that MRI is a feasible and accurate imaging modality for identifying the causes of acute pelvic pain in pediatric females following inconclusive ultrasound examinations. Overall diagnostic performance was high for both non-contrast-enhanced and contrast-enhanced MRI protocols, with excellent sensitivity and specificity across readers with different levels of experience. However, the availability of emergency MRI remains limited in many institutions, particularly outside tertiary referral centers. Therefore, the diagnostic pathway proposed in this study may currently be most applicable to centers with dedicated 24 h MRI availability and experienced radiology teams. Although contrast-enhanced MRI was associated with higher diagnostic confidence, particularly among less experienced readers, this did not translate into a statistically significant improvement in overall diagnostic accuracy compared with non-contrast-enhanced MRI. Nevertheless, performance metrics consistently showed a trend toward higher sensitivity, specificity, and accuracy for contrast-enhanced MRI across readers with different levels of experience. Although these differences did not reach statistical significance, they may still be clinically relevant in selected diagnostic scenarios. These findings suggest that contrast administration may not be routinely required for all patients with acute pelvic pain after inconclusive ultrasound. However, contrast-enhanced MRI may still play an important role as a problem-solving tool in selected cases, particularly when inflammatory complications, active bleeding, or equivocal adnexal findings are suspected.

Gynecological conditions accounted for the majority of positive findings, with hemorrhagic ovarian cysts and adnexal torsion representing the most frequent diagnoses, consistent with previously published data [8,9]. In the case of uterine fibroids, acute pelvic pain was attributed to suspected hemorrhagic or ischemic degeneration, which may present with sudden onset pelvic pain even in adolescent patients. 

**Figure 2:**
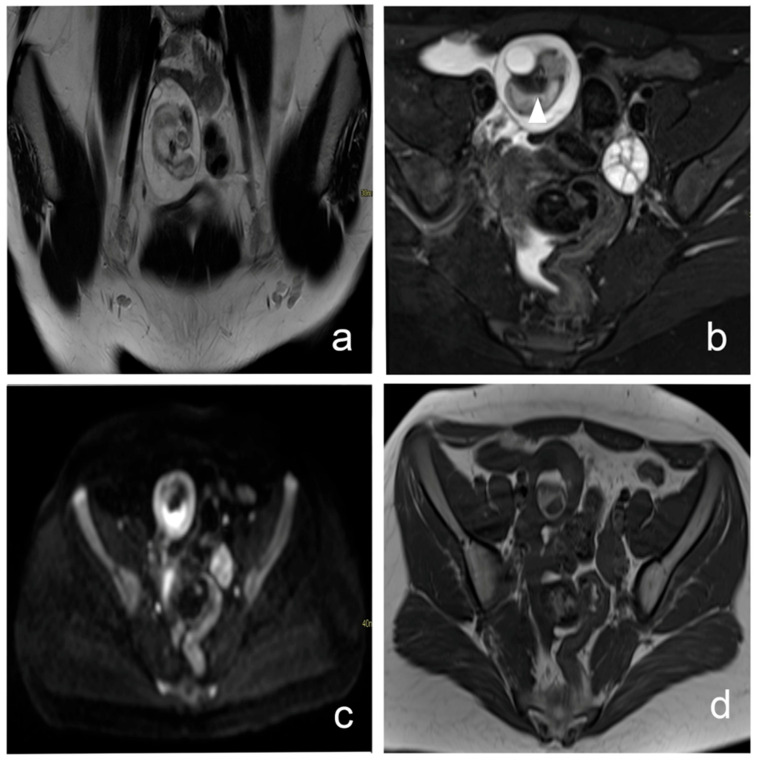
Ovarian torsion associated with a dermoid cyst in a 16-year-old female presenting with acute pelvic pain. (**a**) Coronal T2-weighted HASTE image shows an enlarged right ovary with stromal edema and multiple peripherally displaced follicles. (**b**) Fat-suppressed T2-weighted image shows suppression of the fatty component (head arrow) within the lesion. (**c**) Diffusion-weighted image demonstrates focal diffusion restriction corresponding to the Rokitansky nodule. (**d**) Axial T1-weighted image reveals hyperintense fat-containing components consistent with a dermoid cyst. A twisted vascular pedicle (“whirlpool sign”) and moderate pelvic free fluid are also visible. The presence of ovarian enlargement, stromal edema, and twisted vascular pedicle was consistent with ovarian torsion, which was subsequently confirmed at surgery.

The ability of MRI to accurately detect adnexal torsion is of particular clinical relevance, given the known limitations of ultrasound in this setting. Reported sensitivities for US in diagnosing adnexal torsion are highly variable (51–90.9%) and false-negative examinations may delay surgical management [10,11,12]. MRI, owing to its superior soft-tissue contrast and multiplanar capability, allows detailed assessment of ovarian morphology, stromal edema, hemorrhage, and twisted vascular pedicles, thereby improving diagnostic confidence (Figure 2).

A previous study involving 74 patients reported a sensitivity of 94.6% for MRI in detecting gynecological pathologies, including 100% sensitivity for hemorrhagic cysts [13].

**Figure 3:**
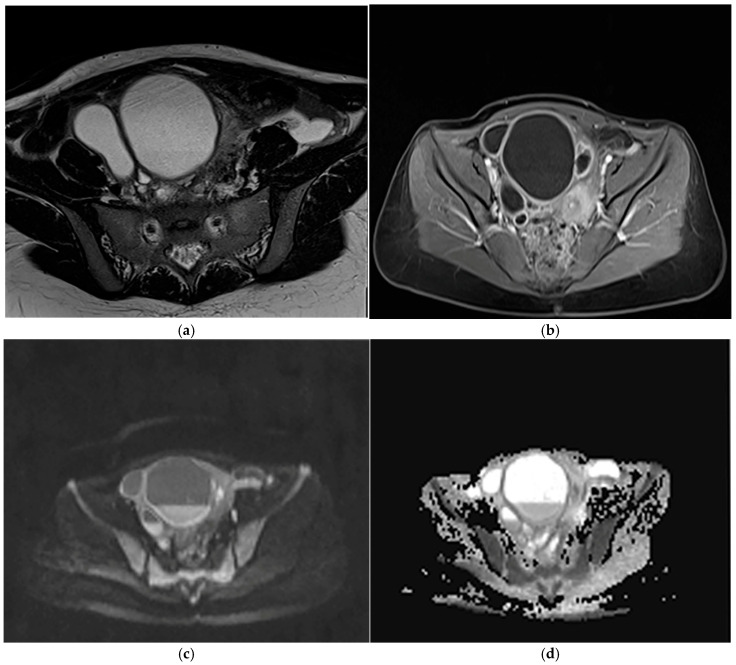
Tubo-ovarian abscess in a 17-year-old female with acute pelvic pain after inconclusive ultrasound. (**a**) Axial T2-weighted HASTE image shows a dilated, convoluted right fallopian tube communicating with a complex right adnexal collection containing fluid–corpuscular material. (**b**) Axial contrast-enhanced T1-weighted image demonstrates thickened and hyperenhancing walls of the collection, consistent with inflammatory changes. (**c**,**d**) Diffusion-weighted image and corresponding ADC map show marked diffusion restriction within the collection, supporting the diagnosis of a tubo-ovarian abscess. The patient was managed conservatively with antibiotic therapy.

Similarly, a prospective study showed the high accuracy of DWI in detecting PID, reporting sensitivity, specificity, PPV, NPV, and overall accuracy of 98.4%, 93.3%, 98.4%, 93.3%, and 97.5%, respectively [14]. A representative case of tubo-ovarian abscess demonstrating restricted diffusion and wall enhancement on MRI is shown in Figure 3.

Beyond gynecological conditions, MRI proved effective in identifying non-gynecological causes of acute pelvic pain, including appendicitis, inflammatory bowel disease, and urinary tract infections. Importantly, a single MRI examination allowed comprehensive evaluation of both pelvic and extra-pelvic structures, reducing the need for additional imaging studies [15].

In our cohort, non-gynecological causes, including intestinal and urological pathologies, made up 25% of the causes of APP. Appendicitis represented the most frequent non-gynecological diagnosis. In these cases, unenhanced MRI correctly identified the condition in all patients included in the study.

**Figure 4:**
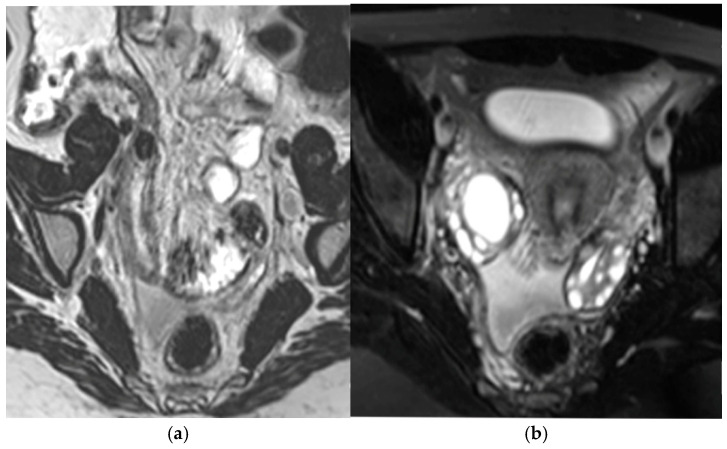
Acute appendicitis in a 15-year-old female presenting with acute pelvic pain. Previous US examination demonstrated a fluid right adnexal cyst, without other significant findings (**a**) Coronal T2-weighted HASTE image shows a tubular structure with thickened walls in the right iliac fossa, containing intraluminal coprolites. (**b**) Axial FS T2-weighted HASTE image confirms the inflamed appendix, with surrounding inflammatory changes and pelvic free fluid. MRI findings were consistent with acute appendicitis and were subsequently confirmed surgically.

A representative case of acute appendicitis detected on MRI is shown in Figure 4.

Our results support previous evidence that MRI may represent a reliable alternative to CT in pediatric patients while avoiding ionizing radiation [16,17].

**Figure 5:**
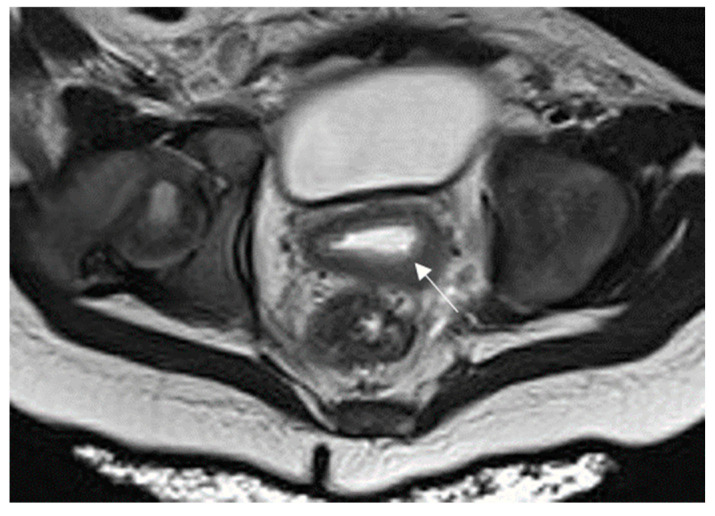
Cervicitis in a 16-year-old female presenting with acute pelvic pain after inconclusive US. Axial T2-weighted image showed cervical enlargement with stromal hyperintensity and wall thickening (arrow). MRI findings, in combination with clinical and laboratory evaluation, supported the diagnosis of cervicitis.

Interobserver and intraobserver agreement were high, particularly among experienced readers, underscoring the reproducibility of MRI interpretation. Most discordant interpretations occurred in subtle inflammatory conditions or small hemorrhagic lesions. For example, the junior reader initially misclassified one case of cervicitis and one case of epiploic appendagitis during the first reading session without contrast administration. Cervicitis may represent a subtle cause of acute pelvic pain and may be challenging to identify on MRI, particularly on unenhanced sequences. A representative case of cervicitis detected on MRI is shown in Figure 5.

**Figure 6:**
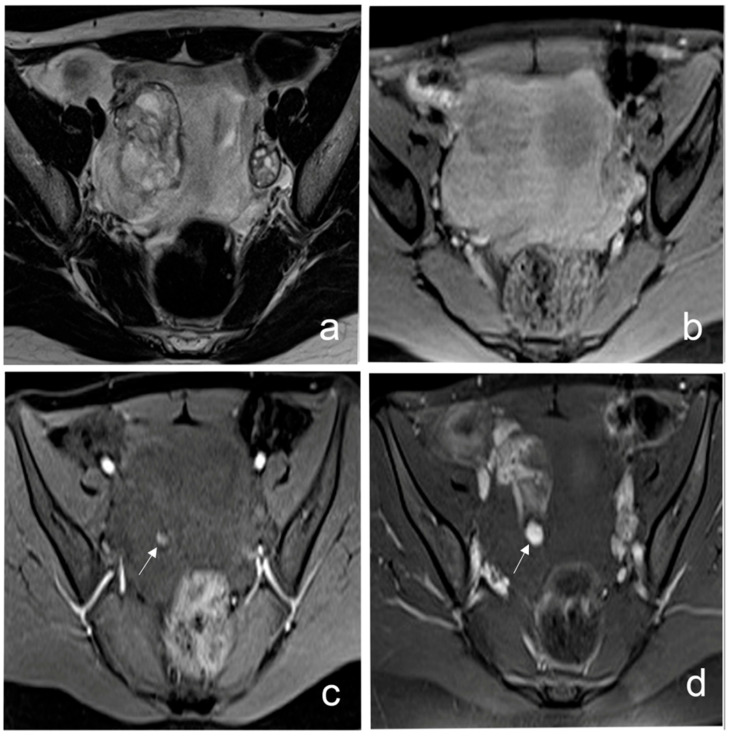
Ruptured hemorrhagic ovarian cyst with hemoperitoneum in a 17-year-old female presenting with acute pelvic pain. (**a**) Axial T2-weighted image shows a right adnexal mass with surrounding hemorrhagic fluid. (**b**) Axial T1-weighted image demonstrates hyperintense hemorrhagic content within the cyst. (**c**,**d**) Axial contrast-enhanced T1-weighted image reveals active contrast extravasation (arrow) into the peritoneal cavity, confirming ongoing bleeding from the ruptured cyst. These findings established the diagnosis of a ruptured hemorrhagic ovarian cyst with hemoperitoneum.

The use of contrast agent was associated with increased diagnostic confidence across all readers, with the greatest benefit observed for the less experienced reader. Nevertheless, non-contrast-enhanced MRI demonstrated strong diagnostic performance, particularly when interpreted by experienced readers, with diffusion-weighted imaging contributing substantially to lesion detection and characterization, particularly in inflammatory conditions and adnexal torsion [18]. The non-contrast-enhanced protocol offers several advantages, including shorter examination times, avoidance of intravenous access, and elimination of potential risks associated with gadolinium-based contrast agents [18,19]. These factors are especially relevant in the pediatric emergency setting, where patient cooperation may be limited. For suspected congenital anomalies, gadolinium is unnecessary, with dedicated MRI protocols focused on uterus-oriented sequences and vaginal evaluation [20]; contrast-enhanced MRI is useful for detecting active bleeding from ruptured adnexal cysts, as shown in Figure 6 [21].

Some studies recently compared the cost of ultrasound (US) versus magnetic resonance imaging (MRI) using time-driven activity-based costing in adolescent female patients with suspected appendicitis and stated that MRI can be a faster and less costly alternative to US for evaluating suspected appendicitis in adolescent female patients [22].

The earlier use of MRI in the emergency department encounter might improve patient throughput and even lower healthcare costs in the long run [22,23].

Recent studies suggested that MRI may be a less costly exam than previously perceived, and perhaps less emphasis should be placed on cost when choosing the appropriate imaging modality for the workup of this particular cohort of patients [22,24].

This study has several limitations. First, by focusing exclusively on patients with inconclusive ultrasound findings, the diagnostic performance of MRI may have been overestimated. Furthermore, the study population was inherently selected, as only clinically stable patients who were able to undergo MRI were included; this selection may limit the generalizability of the results. Second, the use of a composite reference standard including surgical findings, histopathology, and clinical follow-up may introduce verification bias, particularly in patients managed conservatively. This approach reflects routine clinical practice in pediatric emergency settings but may potentially overestimate diagnostic performance. Third, predefined diagnostic categories may have limited flexibility in classifying atypical presentations, although consensus reading was used to mitigate this issue. Additionally, diagnostic performance was evaluated for the identification of the cause of acute pelvic pain as a whole rather than for individual pathologies. Although this approach reflects the clinical diagnostic workflow in emergency settings, the relatively small number of cases for some specific diagnoses did not allow robust statistical analysis for each individual condition. Fourth, unstable or uncooperative patients were excluded, which may further limit the generalizability of the results.

In conclusion, MRI represents an accurate and reliable imaging modality for evaluating acute pelvic pain in pediatric female patients after inconclusive ultrasound. Non-contrast-enhanced MRI provides robust diagnostic information in most pediatric patients with acute pelvic pain after inconclusive ultrasound, while contrast-enhanced imaging may be selectively employed as a problem-solving tool when clinically indicated.

## Figures and Tables

**Table 1:** MRI protocol. DWI EPI MR images were acquired with b values of 50, 500 and 1000 s/mm^2^. Imaging was performed with dynamic volumetric interpolated breath-hold examination with fat saturation; fat saturation was achieved with the chemical-shift-selective fat suppression technique.

Parameter	T2 Half-Fourier Sequence		T2 FSE		T13D Gradient	DWI EPI
	Axial/Axial FS	Coronal	Axial	Sagittal	Axial	Axial
**Repetition time/echo time (ms)**	1000/90	1000/90	4000/85	4000/85	4.1/1.1	2500/80
**Flip angle (°)**	150	150	90	90	10	10
**Field of view (mm)**	320 × 400	320 × 400	260 × 190	260 × 190	320 × 400	280 × 400
**Matrix**	256 × 224	256 × 224	256 × 256	256 × 256	256 × 224	128 × 128
**Parallel imaging factor**	2	2	2	2	3	2
**Section thickness (mm)**	4	4	5	5	2.5	6
**Receiver bandwidth (Hz/pixel)**	62.50	62.50	32	32	62.50	1930
**Intersection gap (mm)**	0	0	1	1	0	0
**NEX**	1	1	2	2	1	6

**Table 2:** Distribution of patients with gynecological diagnosis.

Macro-Category	Specific Cause	*N* (%)
**Bleeding**		23 (38.3%)
	Hemorrhagic cysts	19 (31.6%)
	Ruptured ovarian cysts	4 (6.7%)
**Adnexal torsion**		13 (21.7%)
	Ovarian torsion on cysts	9 (15%)
	Ovarian torsion on teratomas	3 (5%)
	Tubal torsion	1 (1.7%)
**Inflammatory conditions**		5 (8.3%)
	Pelvic inflammatory disease	3 (5%)
	Cervicitis	1 (1.7%)
	Tubo-ovarian abscess	1 (1.7%)
**Masses**		4 (6.7%)
	Uterine fibroids	1 (1.7%)
	Malignancies	1 (1.7%)
	Teratomas	2 (3.3%)

**Table 3:** Distribution of patients with non-gynecological diagnosis.

**Gastrointestinal**	**Inflammatory conditions**		10 (16.7%)
		Appendicitis	6 (10.0%)
		Crohn’s disease	1 (1.7%)
		Ulcerative colitis	1 (1.7%)
		Meckel diverticulitis	1 (1.7%)
		Epiploic appendagitis	1 (1.7%)
	**Obstruction**		2 (3.3%)
**Urological**	**Inflammation**	Pyelonephritis	2 (3.3%)
**Musculoskeletal**	**Inflammation**	Pubic osteomyelitis	1 (1.7%)

**Table 4:** Performance of unenhanced MRI in diagnosing acute pelvic pain in pediatric patients.

	Sensitivity (%)	Specificity (%)	Positive Predictive Value (%)	Negative Predictive Value (%)	Accuracy (%)
**Senior reader**	88.33 (77.43, 95.18)	78.57( 59.05, 91.70)	89.83 (81.20, 94.75)	75.86 (60.41, 86.62)	85.23 (76.06, 91.89)
**Intermediate reader**	76.67 (63.96, 86.62)	78.57 (59.05, 91.70)	88.46 (78.82, 94.05)	61.11 (48.86, 72.11)	77.27 (67.11, 85.53)
**Junior reader**	75.00 (62.14, 85.28)	64.29 (44.07, 81.36)	81.82 (72.83, 88.31)	54.55 (41.69, 66.83)	71.59 (60.98, 80.70)

Values are expressed as percentages, with 95% confidence intervals reported in parentheses.

**Table 5:** Performance of MRI with contrast in diagnosing acute pelvic pain in pediatric patients.

	Sensitivity (%)	Specificity (%)	Positive Predictive Value (%)	Negative Predictive Value (%)	Accuracy (%)
**Senior reader**	95 (86.08, 98.96)	90 (73.47, 97.89)	95.00 (86.64, 98.24)	90.00 (74.80, 96.47)	93.33 (86.05, 97.51)
**Intermediate reader**	85 (73.43, 92.90)	85.71 (67.33, 95.97)	92.73 (83.64, 96.95)	72.73 (58.90, 83.23)	85.23 (76.06, 91.89)
**Junior reader**	81.67 (69.56, 90.48)	85.71 (67.33, 95.97)	92.45 (83.07, 96.83)	68.57 (55.60, 79.17)	82.95 (73.45, 90.13)

Values are expressed as percentages, with 95% confidence intervals reported in parentheses.

## Data Availability

The data presented in this study are not publicly available due to ethical and privacy restrictions related to pediatric patient data. De-identified imaging and clinical data may be made available from the corresponding author upon reasonable request, subject to institutional approval.

## Abbreviations

The following abbreviations are used in this manuscript:

APP	acute pelvic pain
US	ultrasound
MRI	magnetic resonance imaging
FSE	Fast Spin Echo
FISP	Fast Imaging with steady-state precession
DWI	Diffusion-Weighted Imaging
EPI	Echo-Planar Imaging
FS	Fat Saturation
NEX	number of excitations
